# Socioeconomic Gradient in Childhood Obesity and Hypertension: A Multilevel Population-Based Study in a Chinese Community

**DOI:** 10.1371/journal.pone.0156945

**Published:** 2016-06-03

**Authors:** Patrick Ip, Frederick Ka Wing Ho, Hung-Kwan So, Dorothy Fung-ying Chan, Matthew Ho, Winnie Tso, E. Anthony S. Nelson

**Affiliations:** 1 Department of Paediatrics and Adolescent Medicine, The University of Hong Kong, Hong Kong, China; 2 Department of Paediatrics, The Chinese University of Hong Kong, Hong Kong, China; University Children's Hospital Tuebingen, GERMANY

## Abstract

**Background:**

This study aims to assess evidence for any socioeconomic gradients in childhood obesity and hypertension in a population-representative sample in Hong Kong, China.

**Methods:**

The data of a stratified random sampled growth survey collected in 2005–2006 was matched with a population by-census. Obesity was defined using the International Obesity Task Force standard and hypertension was defined using the Hong Kong norm table. Family socioeconomic status (SES) was measured by maternal education level. Neighbourhood SES was measured by median household income of the neighbourhood. Multilevel Poisson regression models with robust standard error were used to test the association. Body mass indices of children’s parents were included as potential confounders. Intra-school/neighbourhood correlations were adjusted using random factors.

**Results:**

Totally 14842 children (age 6–19 years) included in the analysis, in which 16.6% of them were overweight or obese. Children whose mother only completed secondary school or below had higher risk of childhood obesity (RR 1.41, 95% CI 1.13–1.76, p = 0.003) and hypertension (RR 1.18, 95% CI 1.01–1.36, p = 0.03). Meanwhile, children in the lowest neighbourhood SES group had higher risk of childhood underweight (RR 1.61, 95% CI 1.04–2.49, p = 0.03), overweight (RR 1.35, 95% CI 1.05–1.72, p = 0.02), and obesity (RR 2.07, 95% CI 1.11–3.88, p = 0.02).

**Conclusions:**

Socioeconomic gradient in childhood obesity and hypertension existed in Hong Kong, one of the most developed cities in China. These results have implications for policymakers and public health experts and highlight the need to monitor trends in other parts of China.

## Introduction

Childhood obesity is an important public health issue associated with long term morbidity such as adulthood obesity, hypertension and cardiovascular disease [1–3]. Along with the rapid urbanization and changing lifestyles in developing countries, childhood obesity is expected to worsen in countries like China. As the cause of childhood obesity is multifactorial, an ecological framework has been proposed to improve our understanding of its etiology [4]. The framework suggests that childhood obesity is a direct consequence of positive energy imbalance related to improper nutrition and exercise behaviour, which are influenced by an array of multilevel social factors including family and neighbourhood socioeconomic status (SES).

However, the relationship between SES and childhood obesity is complex and varies with culture [5, 6]. Lower SES is generally found to have be associated with childhood overweight and obesity in Western developed countries, including the US, United Kingdom and Germany [7]. However the association between childhood obesity and low SES is less clear in other parts of the world. For example, no significant relationship was observed between family affluence and overweight in Russia [8], and Chinese children from wealthier families were reported to have a higher risk of obesity [6]. A preliminary study conducted 15 years ago in Hong Kong, a rapidly developing city in Southern China, identified no association between childhood overweight/obesity and a set of SES parameters, such as parental education level and family income [9]. This mixed evidence calls for a more robust and up-to-date assessment of this important public health issue within the Chinese context, especially given the rapid economic growth of China in the recent two decades.

Although obesity and hypertension are closely related, the social determinants of blood pressure (BP) are less well explored. Previous assessments often focused on associations in adulthood, which identified inverse associations between SES and BP of adults in both Western developed countries and urban China [10, 11]. Whether these findings, and to what extent, are applicable to children requires further study.

We analysed data collected in the Hong Kong Growth Study 2005–2006 (HKGS), combined with census data, with the following aims: 1. To study the association between family and school neighbourhood-level SES and childhood underweight, overweight, obesity, and hypertension; 2. To estimate the population attributable fraction of family and school neighbourhood SES; 3. To investigate whether two well-studied factors contributing to overweight and obesity (breakfast and exercise frequency) mediate the identified relationships.

## Methods

This study utilised data from the HKGS 2005–2006 and the Population By-census 2006. The Growth Study was a two-stage stratified cluster random sampling study collecting children’s anthropometric parameters, BP, and family sociodemographic data. Details of the sampling frame have been previously reported.[12, 13] The Population By-census was a multistage stratified random sampling survey conducted by the Hong Kong Census and Statistics Department with rigorous quality control procedures [14].

### Participants

A list of schools registered in the Hong Kong Education Bureau was complied. Two schools (one primary and one secondary) were randomly selected from each of the 18 administrative districts in Hong Kong. Two classes in each grade were then randomly selected from the participating schools, in which all students were invited to participate in this study. The parents of all participants were invited to complete a questionnaire providing demographic information that included gestation, birth weight, feeding pattern and family or personal history of obesity risk factors. The overall response rate was 91% (93% in primary and 90% in secondary school). More than two-thirds of the non-response were due to absence from school on the testing day.

### Measurements

#### Anthropometric parameter and blood pressure

Students’ anthropometric parameters and blood pressure were collected in the HKGS. Eight trained research staff visited all participating schools to collect students’ anthropometric parameters. Standing height without shoes was measured using a Harpenden Stadiometer to the nearest 1 mm. Body weight was measured using a Tanita scale (Model BF-522). Blood pressure was measured at school in the morning using the Datascope Accutorr Plus at the right arm. The students were seated and rested with their right arm supported at heart level for at least five minutes before measurement. Cuffs with at least 40% of arm circumference, length 80 to 100% of arm circumference were used [15]. Two measurements of blood pressure were taken with a one-minute interval and the average was used in analysis.

Body mass index (BMI) was calculated by dividing the weight (in kg) by the square of height (in m). Underweight, overweight, and obesity was defined using the International Obesity Task Force (IOTF) age- and sex-specific cut-off values [16]. Hypertension was defined as those systolic blood pressure and/or diastolic blood pressure greater than or equal to sex-, age-, and height-specific 95^th^ percentile or 140/90 mmHg [12].

#### Family socioeconomic status

Each participant’s parental education levels (1. primary school or below, 2. secondary school, 3. post-secondary school certificate/diploma, or 4. bachelor degree or above) were self-reported in the family questionnaire of the HKGS. Only maternal education level was included in the analysis because of the high correlation between the maternal and paternal education level (81.1% agreement). Family income was not asked in this study.

#### Neighbourhood socioeconomic status

School neighbourhood SES was represented by the tertiary planning unit (TPU) level median monthly household income as reported from the Hong Kong Population By-census 2006. TPU is an administrative geographical unit in Hong Kong and there were 204 TPUs in 2006. To avoid linearity assumption, neighbourhood income was categorised to four groups according to quartiles (≤ HKD 14654 [Low], > HKD 14654 to HKD 17625 [Lower middle], > HKD 17625 to HKD 25000 [Upper middle], and > HKD 25000 [High]).

#### Potential Mediators

Two potential mediators related to physical activity (PA) and nutrition were asked in the family questionnaire of the HKGS. The PA-related potential mediator was the number of exercise class other than school physical education lessons in a week. This question was designed based on the physical activity guidelines for adolescents published in the international consensus conference [17]. An explanation and a detailed list of common exercise classes in Hong Kong were provided in the questionnaire. The item has been shown to have good predictive validity in previous reports [18]. Number of school physical education lessons was consistent across schools (two 45-minute lessons per week [19]) so it was not considered in this study. The nutrition-related potential mediator was the number of days per week that the child ate breakfast. Breakfast frequency was selected because it has been consistently reported to have a negative association with childhood obesity [20, 21]. This item was previously validated against a seven days of 24-hour dietary recall study of 391 Hong Kong Chinese students with the percentage of agreement from 72.9–90.8% [22].

#### Potential confounders

Parents’ weight status may affect both family SES and children’s weight and hypertension status, so their parents' BMIs were regarded as potential confounders in this study. Parents’ height and weight were self-reported in the family questionnaire of the HKGS and BMIs were calculated using the previously stated formula.

### Statistical analysis

Generalised linear mixed models were used to investigate the SES variables associate with body weight and BP status. Underweight, overweight, obese, and hypertensive were entered into the model as binary dependent variables. Maternal education level and neighbourhood income level were the primary independent variables of interest. Both parents’ BMIs and children’s age and gender were included as covariates. School/neighbourhood variations were captured random factors to reflect the sampling frame. Despite the binary outcome variables, Poisson distribution with logarithm link function was used so that risk ratio (relative risk) could be estimated. The under-dispersion that arouse from fitting binary outcome variable with Poisson distribution was corrected using a robust standard error estimator for estimating accurate p-values [23]. Because parents’ BMIs may not be confounders as we hypothesised, a sensitivity analysis was conducted to understand whether excluding these two variables would affect the results of our analysis.

The contribution of SES to childhood weight status and hypertension at population level was quantified using population attributable fractions using standard formulae [24]. The proportion of children in different SES categories (i.e. proportion of exposure) was directly estimated in this study. Three hypothetical scenarios were constructed to understand how the prevalence of weight and hypertension problem might change under social interventions: 1) increasing education opportunity (the prevalence of maternal tertiary education increased by 25%); 2) overall economic improvement (25% of children from each of the low, lower middle, and upper middle SES neighbourhood shifted to the lower middle, upper middle, and high SES neighbourhood respectively); 3) a poverty reduction programme (75% of children from low SES neighbourhood shifted to lower middle, and 50% of children from lower middle SES neighbourhood shifted to upper middle). The proportional reduction in weight and hypertension problems for these three scenarios were estimated accordingly [24].

The role of potential mediators on hypertension and obesity was analysed under the causal mediation analysis framework [25]. The framework is a generalisation of the commonly-used Baron and Kenny approach [26], which has relaxed assumptions and is applicable to binary outcomes with multilevel structure. A general overview of the framework can be found in Imai et al’s paper [25]. The approach can be summarised in four steps: 1. identify the associations between health outcomes and SES, which were performed in the previous regression analysis; 2. estimate the effect of the SES variables on the two mediators; 3. estimate the effect of the mediators on the health outcomes after controlling for SES; and 4. estimate the mediated effect and the proportion mediated. All the above steps were carried out using hierarchical models to control for confounders and intra-school/neighbourhood correlation.

All analyses were performed using *R Statistical Package* version 3.2.1 with packages *lme4* [27] and *mediation* [28].

### Ethics

The study was approved by the Joint Chinese University of Hong Kong and New Territories East Cluster Clinical Research Ethics Committee and the Ethics Committee of the Hong Kong Department of Health.

## Results

### Participant characteristics

14842 primary and secondary school students (age 6–19 years, mean = 12.07, all are Chinese) were recruited in this study. Female-to-male ratio was 1:0.99. Their BMI z-score had a mean of 0.11 (SD 1.15). Using the IOTF criteria, 16.6% of the students were overweight or obese, and 3.6% were underweight. Their average blood pressure was 109.2/64.6 mmHg (SD 12.6/9.5 mmHg) and 1216 (8.19%) of the students had hypertension. The majority (62.0%) of students did not participate in any exercise in addition to their regular physical education lessons. Two-third of the students are breakfast at least six days a week and 5.1% skipped all breakfasts. Fifty-three percent of the students’ mothers had completed only primary or secondary education. The majority of participants’ (47.2%) school neighbourhood SES fell into the lower middle group (HKD > 14654 to HKD 17625). Detailed participant characteristics are shown in Table 1.

**Table 1:** Participant characteristics.

	Mean (SD) / Number (%)
Age, mean (SD), years	12.07 (3.47)
Gender	
Female	7370 (49.7%)
Male	7472 (50.3%)
BMI z-score, mean (SD)	0.11 (1.15)
Weight status	
Underweight	537 (3.6%)
Normal	11838 (79.8%)
Overweight	1905 (12.8%)
Obese	562 (3.8%)
Systolic blood pressure, mean (SD), mmHg	109.15 (12.57)
Diastolic blood pressure, mean (SD), mmHg	64.63 (9.47)
Hypertension, n (%)	1216 (8.19%)
Maternal education level	
Secondary school or below	6015 (52.9%)
Tertiary education or above	5363 (47.1%)
Neighbourhood median monthly income, HKD	
≤ 14654	2246 (15.1%)
> 14654 to 17625	7004 (47.2%)
> 17625 to 25000	4202 (28.3%)
> 25000	1390 (9.4%)
Exercise frequency, number of times per week	
None	6517 (62.0%)
Once	1923 (18.3%)
Twice	1181 (11.2%)
Three times or more	893 (8.5%)
Number of days having breakfast	
None	582 (5.1%)
1 to 3	1375 (12.2%)
4 to 5	1805 (16.0%)
6 to 7	7536 (66.7%)
Paternal BMI, mean (SD), kg/m^2^	23.58 (3.75)
Maternal BMI, mean (SD), kg/m^2^	22.03 (3.52)

### Association between SES and weight/hypertension status

**Fig 1:**
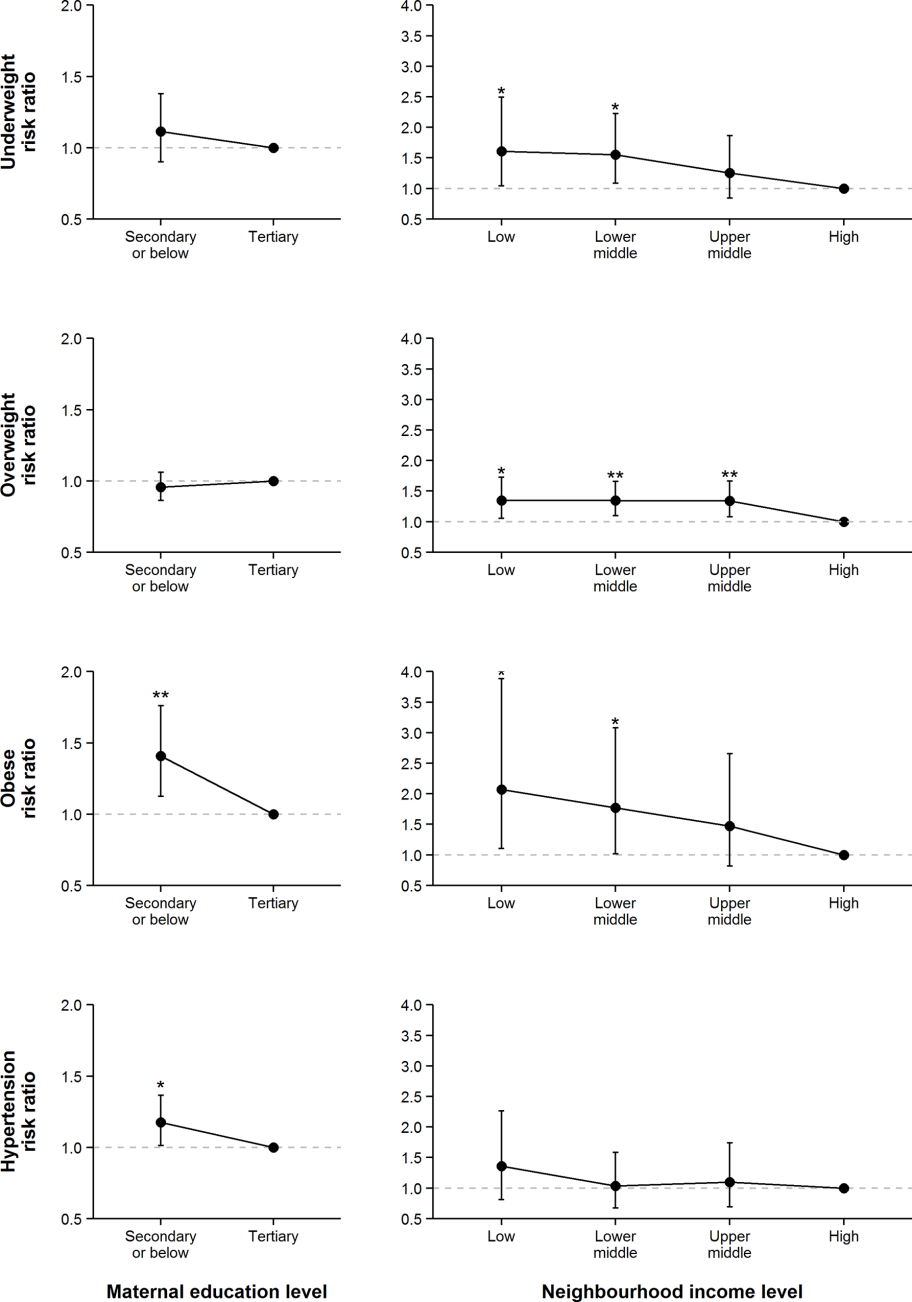
Adjusted associations between SES and weight and hypertension status. All associations were estimated after the adjustment of child’s gender and age, paternal and maternal BMI, and intra-cluster correlation. Tertiary maternal education and low neighbourhood income were the reference groups. Error bars show the 95% confidence intervals. **: p < 0.01; *: p < 0.05

Associations between SES and weight/hypertension status are shown in Fig 1. After controlling for potential confounders, children whose mother only completed secondary school or below had a higher risk of childhood obesity (RR 1.41, 95% CI 1.13–1.76, p = 0.003) and hypertension (RR 1.18, 95% CI 1.01–1.36, p = 0.03). Children in the lowest neighbourhood SES group had a higher risk of underweight (RR 1.61, 95% CI 1.04–2.49, p = 0.03), overweight (RR 1.35, 95% CI 1.05–1.72, p = 0.02), and obesity (RR 2.07, 95% CI 1.11–3.88, p = 0.02). These associations were not significantly moderated by gender and age group (p = 0.12–0.89). Sensitivity analysis also found no discrepancy in association pattern if parents’ BMI were not adjusted in the models (S1 Table).

The contribution of SES to weight and hypertension morbidities at a population level is presented in Table 2. Maternal education level accounted for 17.8% and 8.5% of the population’s childhood obesity and hypertension respectively, while neighbourhood income level accounted for 29.8%, 23.8% and 39.7% of the population’s childhood underweight, overweight, and obesity respectively. A 25% higher prevalence in maternal tertiary education is associated with 4.4% and a 2.1% lower prevalence in obesity and hypertension. The overall economic development scenario is associated with 3.9%, 1.9%, and 4.8% lower prevalence in underweight, overweight, and obesity. Furthermore, a poverty reduction programme (75% of children from low SES neighbourhood shifted to lower middle, and 50% of children from lower middle SES neighbourhood shifted to upper middle) scenario is associated with 5.5%, 0.2%, and 6.3% lower prevalence of underweight, overweight, and obesity respectively.

**Table 2:** Prevalence and population attributable fractions of weight and hypertension problems by SES.

	Underweight	Overweight	Obesity	Hypertension
**Prevalence by maternal education level, %**				
Secondary school or below	4.14	17.15	3.84	8.56
Tertiary education or above	3.34	15.03	3.00	7.55
**Prevalence by neighbourhood income level, %**				
≤ HKD 14654	3.52	17.72	4.94	9.17
> HKD 14654 to 17625	3.95	16.66	3.77	7.75
> HKD 17625 to 25000	3.19	17.87	3.86	8.83
> HKD 25000	3.38	10.86	1.80	6.91
**Population attribution fraction**				
Maternal education level	-	-	17.8%	8.5%
Neighbourhood income level	29.8%	23.8%	39.7%	-
**Proportional reduction in hypothetical scenarios**				
Increased opportunity in tertiary education	-	-	4.4%	2.1%
Overall economic development	3.9%	1.9%	4.8%	-
Poverty reduction programme	5.5%	0.2%	6.3%	-

### Mediation between SES and obesity/hypertension

Table 3 shows the results of the mediation analysis. Maternal tertiary education was found to have a positive association with both breakfast (β 0.14; 95% CI 0.05–0.23; p = 0.002) and exercise (β 0.14; 95% CI 0.10–0.18; p = 0.002) frequency. After controlling for maternal education level, higher breakfast frequency was associated with lower risk of obesity (OR 0.88; 95% CI 0.84–0.92; p < 0.001) but not with the risk of hypertension (OR 1.02; 95% CI 0.99–1.06; p = 0.24); frequent exercise was associated with lower risks of obesity (OR 0.86; 95% CI 0.76–0.97; p = 0.01) and hypertension (OR 0.85; 95% CI 0.78–0.92; p < 0.001).

**Table 3:** Association between health outcomes and potential mediator.

Dependent variable	Independent variable	Coefficient / odds ratio (95% CI)	p
**Step 2**^**a**^			
Breakfast frequency	Maternal tertiary education	0.14 (0.05 to 0.23)	0.002
Breakfast frequency	Neighbourhood income	0.05 (-0.05 to 0.16)	0.31
Exercise frequency	Maternal tertiary education	0.14 (0.10 to 0.18)	< 0.001
Exercise frequency	Neighbourhood income	0.03 (-0.03 to 0.08)	0.32
**Step 3**^**b**^			
Obesity	Breakfast frequency	0.88 (0.84 to 0.92)	< 0.001
Obesity	Exercise frequency	0.86 (0.76 to 0.97)	0.01
Hypertension	Breakfast frequency	1.00 (0.97 to 1.03)	0.93
Hypertension	Exercise frequency	0.85 (0.78 to 0.92)	< 0.001

^a^ Regressing SES variables on mediators

^b^ Regressing mediators on health outcomes while controlling for maternal education level

All associations were estimated after the adjustment of child’s gender and age, paternal and maternal BMI, and intra-cluster correlation.

Table 4 shows the mediated effects and proportions. The effect of maternal education level on obesity can be explained by breakfast (proportion mediated 3.1%) and exercise (proportion mediated 4.3%) frequency, while the effect of maternal education on hypertension can only be explained by exercise frequency (proportion mediated 16.4%).

**Table 4:** Mediation between health outcomes and maternal tertiary education.

	Mediated effect (95% CI) ^a^	P	Proportion mediated	Controlled direct effect (95% CI) ^a^	P
**Obesity**					
Breakfast frequency	-0.0004 (-0.0008 to -0.0001)	***	3.1%	-0.0117 (-0.0190 to -0.0042)	***
Exercise frequency	-0.0005 (-0.0010 to -0.00004)	*	4.3%	-0.0120 (-0.0200 to -0.0048)	***
**Hypertension**					
Breakfast frequency	-0.0002 (-0.0040 to 0.0190)		2.3%	-0.0076 (-0.0190 to -0.0040)	*
Exercise frequency	-0.0020 (-0.0030 to -0.0007)	***	16.4%	-0.0067 (-0.0185 to 0.0054)	

^a^ Estimated using quasi-Bayesian method after the adjustment of child’s gender and age, and intra-cluster correlation.

***: P<0.001

*: P<0.05

## Discussion

This study has found that lower family SES was associated with higher risk in childhood obesity and hypertension, whereas lower neighbourhood SES was associated with higher risk in childhood underweight, overweight, and obesity. These socioeconomic factors appeared to have a significant contribution to the population’s weight and hypertension problems, in which breakfast and exercise acted as the partial mediators.

The identified inverse relationship between SES and childhood obesity was indeed consistent with that found in Western developed countries. A systematic review [7] has identified a general inverse relationship between parental educational attainment and children’s obesity with equivalent risk ratios between 1.84 and 2.25, slightly higher that what we have found (1.41) in this study. Nevertheless, these results may not be applicable to other parts of the world. A study utilising 20 years (1985–2005) of national survey data from the Mainland China (not including Hong Kong) has identified an overall rise in childhood overweight and obesity prevalence in the whole territory, but the increasing trend was not at the same pace [29]. The rise of overweight and obesity prevalence was much more prominent in the wealthier urban regions than the poorer rural areas. Such phenomenon was also found in other developing countries, where fatness of children is a symbol of affluence and poorer children are often struggling with malnutrition [30]. However, we should note that the Chinese study was conducted 10 years ago and the situation may have changed as a result of the rapid economic development in China in the past decade. In the wealthier parts of China where malnutrition and underweight are rare, the underprivileged children may encounter an elevated risk of overweight and obesity, just like Hong Kong.

In fact, we should beware that the health disparity was not only found in overweight, obesity, and hypertension, but also in underweight. Although the prevalence of childhood underweight in Hong Kong was rather low (3.6%), policymakers and healthcare professionals should recognise the double jeopardy of children from lower SES neighbourhood. Underweight may be an indicator of malnutrition and, even at its milder form (-1 to -2 SD of weight-for-age), it has strong associations with higher mortality from diarrhoea, pneumonia, malaria, and measles [31]. Moreover, malnutrition may also hinder the optimal development of children [32].

One nutrition- and one exercise-related factor were considered in this study and both were found to mediate the health disparities in children, which was consistent with previous reports [33, 34]. In fact, the two mediators selected not only represent the actual frequency of breakfast and exercise, but may also reflect the general health literacy of the family, as well as the accessibility to health lifestyle. Having a higher socioeconomic background (such as better parental education, family wealth, and income) not only provides the necessary resources for children to thrive, but also the important health-related consciousness and literacy among parents to promote healthy behaviours among their children [35, 36]. Therefore, parents with lower educational attainment may not be able to recognise what is a suitable healthy lifestyle for their children to prevent obesity and hypertension. Furthermore, healthy lifestyle in a well-developed city is often associated with higher cost. It is well known that energy-dense food without much other nutrients (i.e. food with ‘empty calories’) often cost much less than those nutrient-dense food (such as lean meat and fresh vegetables) [37]. This provides an obvious financial incentive for parents to choose less healthy foods for their children. In fact, the accessibility to healthy lifestyle not only operate on individual level but also on a school neighbourhood level. A recent multilevel study in Hong Kong has found that the schools in upper class regions often had a larger campus size (r = 0.11, p = 0.02), providing the necessary space for their students to exercise, while some primary schools in poorer regions of Hong Kong did not even have a full basketball court [38].

The identified disparities in childhood underweight, obesity and hypertension calls for a more fundamental approach for these important public health issues. A few hypothetical social interventions were examined in this study. Even though the estimated prevalence reduction was modest, there are still opportunities for relevant improvements to make at population level to alleviate factors increasing disease burden. Moreover, we should note that social interventions not only provide improvements to children’s weight and BP problems, but also to all SES-related morbidity and mortality and associated productivity loss [39, 40]. These health and health-related effects of social interventions should be well recognised and considered by policymakers.

Findings of the current study should be interpreted with the following caveats. First, the child growth survey was originally conducted in 2006, and with the recent rise of economic disparity in the region [41], the health disparities could have worsened. Second, this is a cross-sectional study in which causality cannot be confirmed. However, it was unlikely that children’s obesity and hypertension would affect their family and neighbourhood SES. Third, there were only two mediators included in this study and the mediation proportion of these two factors were small. Further studies should consider more potential mediators in Chinese populations, especially on healthy lifestyle-related literacy and accessibility. Fourth, the current study could not identify the reasons why some low SES areas were associated with both underweight and obesity. Future investigation should collect data on the town planning level (e.g. walkability, land use for fresh markets) to understand this issue better.

The present study provides robust evidence to illustrate the health disparity in childhood underweight, overweight, obesity and hypertension. A significant proportion of the prevalence could be attributable to socioeconomic disparity. Further investigations on the upstream factors (e.g. reducing socioeconomic inequalities) should be conducted to inform the impact of social interventions on population health.

## Supporting Information

S1 Table Sensitivity analysis on the association between SES and weight and hypertension status. All associations were estimated after the adjustment of child’s gender and age, and intra-cluster correlation. Error bars show the 95% confidence intervals. **: p < 0.01; *: p < 0.05
(XLSX)

## References

[1] Lauer R, Clarke W. Childhood risk factors for high adult blood pressure: the Muscatine Study. Pediatrics. 1989;84(4):633–41. 2780125

[2] Freedman DS, Khan LK, Dietz WH, Srinivasan SR, Berenson GS. Relationship of childhood obesity to coronary heart disease risk factors in adulthood: the Bogalusa Heart Study. Pediatrics. 2001;108(3):712–8. 11533341 10.1542/peds.108.3.712

[3] Arnett DK, Glasser SP, McVeigh G, Prineas R, Finklestein S, Donahue R, et al Blood pressure and arterial compliance in young adults: the Minnesota Children's Blood Pressure Study. Am J Hypertens. 2001;14(3):200–5. 11281229 10.1016/s0895-7061(00)01262-0

[4] Davison KK, Birch LL. Childhood overweight: a contextual model and recommendations for future research. Obes Rev. 2001;2(3):159–71. 12120101 10.1046/j.1467-789x.2001.00036.x PMC2530932

[5] Stamatakis E, Wardle J, Cole TJ. Childhood obesity and overweight prevalence trends in England: evidence for growing socioeconomic disparities. Int J Obes. 2010;34(1):41–7. 10.1038/ijo.2009.217 PMC3865596 19884892

[6] Wang Y. Cross-national comparison of childhood obesity: the epidemic and the relationship between obesity and socioeconomic status. Int J Epidemiol. 2001;30(5):1129–36. 11689534 10.1093/ije/30.5.1129

[7] Shrewsbury V, Wardle J. Socioeconomic Status and Adiposity in Childhood: A Systematic Review of Cross‐sectional Studies 1990–2005. Obesity. 2008;16(2):275–84. 10.1038/oby.2007.35 18239633

[8] Due P, Damsgaard MT, Rasmussen M, Holstein BE, Wardle J, Merlo J, et al Socioeconomic position, macroeconomic environment and overweight among adolescents in 35 countries. Int J Obes. 2009;33(10):1084–93. 10.1038/ijo.2009.128 PMC3421462 19621018

[9] Hui L, Nelson E, Yu L, Li A, Fok T. Risk factors for childhood overweight in 6-to 7-y-old Hong Kong children. Int J Obes. 2003;27(11):1411–8. 10.1038/sj.ijo.0802423 14574354

[10] Grotto I, Huerta M, Sharabi Y. Hypertension and socioeconomic status. Curr Opin Cardiol. 2008;23(4):335–9. 10.1097/HCO.0b013e3283021c70 18520717

[11] Yu Z, Nissinen A, Vartiainen E, Song G, Guo Z, Zheng G, et al Associations between socioeconomic status and cardiovascular risk factors in an urban population in China. Bull WHO. 2000;78(11):1296–305. 11143189 PMC2560638

[12] Sung RY, Choi KC, So H-K, Nelson EA, Li AM, Kwok CW, et al Oscillometrically measured blood pressure in Hong Kong Chinese children and associations with anthropometric parameters. J Hypertens. 2008;26(4):678–84. 10.1097/HJH.0b013e3282f42270 18327076

[13] So H-K, Nelson E, Li A, Wong E, Lau J, Guldan G, et al Secular changes in height, weight and body mass index in Hong Kong Children. BMC Public Health. 2008;8(1):320. 18803873 10.1186/1471-2458-8-320 PMC2572616

[14] Census and Statistics Department of Hong Kong SAR Government. Basic Tables for Tertiary Planning Units. Hong Kong: 2007.

[15] National High Blood Pressure Education Program. Update on the 1987 Task Force Report on High Blood Pressure in Children and Adolescents: A Work Group Report from the National High Blood Pressure Education Program. Pediatrics. 1996;98:649–57. 8885941

[16] Cole T, Lobstein T. Extended International (IOTF) Body Mass Index Cut‐Offs for Thinness, Overweight and Obesity. Pediatr Obes. 2012;7(4):284–94. 10.1111/j.2047-6310.2012.00064.x 22715120

[17] Sallis JF, Patrick K. Physical activity guidelines for adolescents: Consensus statement. Pediatr Exerc Sci. 1994;6:302–.

[18] So H, Sung R, Li A, Choi K, Nelson E, Yin J, et al Higher exercise frequency associated with lower blood pressure in Hong Kong adolescents: a population-based study. Journal of human hypertension. 2010;24(10):646–51. 10.1038/jhh.2009.117 20090774

[19] Hui S. Health and physical activity in Hong Kong. Hong Kong: Hong Kong Sports Development Board, 2001.

[20] Rampersaud GC, Pereira MA, Girard BL, Adams J, Metzl JD. Breakfast habits, nutritional status, body weight, and academic performance in children and adolescents. J Am Diet Assoc. 2005;105(5):743–60. 15883552 10.1016/j.jada.2005.02.007

[21] So H, Nelson E, Li AM, Guldan G, Yin J, Ng P, et al Breakfast frequency inversely associated with BMI and body fatness in Hong Kong Chinese children aged 9–18 years. Br J Nutr. 2011;106(05):742–51. 21535905 10.1017/S0007114511000754

[22] Guldan GS, Lau TS, Lee HM. Validity and reliability of a Two-Minute Assessment rapid dietary questionnaire measuring healthy eating behaviours among Hong Kong primary school students. Hong Kong Med J. 2014;20(Suppl 7):30–3. 25647823

[23] Zou G. A modified poisson regression approach to prospective studies with binary data. Am J Epidemiol. 2004;159(7):702–6. 15033648 10.1093/aje/kwh090

[24] Rockhill B, Newman B, Weinberg C. Use and misuse of population attributable fractions. Am J Public Health. 1998;88(1):15–9. 9584027 10.2105/ajph.88.1.15 PMC1508384

[25] Imai K, Keele L, Tingley D, Yamamoto T. Unpacking the black box of causality: Learning about causal mechanisms from experimental and observational studies. Am Polit Sci Rev. 2011;105(04):765–89.

[26] MacKinnon DP. Mediation analysis In: Cautin RL, Lilienfeld SO, editors. The Encyclopedia of Clinical Psychology. New York: Wiley-Blackwell; 2008.

[27] Bates D, Mächler M, Bolker B, Walker S. Fitting linear mixed-effects models using lme4. arXiv e-prints. 2014;1406.5823v1 [stat.CO].

[28] Tingley D, Yamamoto T, Hirose K, Keele L, Imai K. Mediation: R package for causal mediation analysis. J Stat Softw. 2014;59(5).

[29] Ji CY, Cheng TO. Epidemic increase in overweight and obesity in Chinese children from 1985 to 2005. Int J Cardiol. 2009;132(1):1–10. 10.1016/j.ijcard.2008.07.003 18835050

[30] Prentice AM. The emerging epidemic of obesity in developing countries. Int J Epidemiol. 2006;35(1):93–9. 16326822 10.1093/ije/dyi272

[31] Caulfield LE, de Onis M, Blössner M, Black RE. Undernutrition as an underlying cause of child deaths associated with diarrhea, pneumonia, malaria, and measles. Am J Clin Nutr. 2004;80(1):193–8. 15213048 10.1093/ajcn/80.1.193

[32] Engle PL, Black MM, Behrman JR, de Mello MC, Gertler PJ, Kapiriri L, et al Child development in developing countries 3: Strategies to avoid the loss of developmental potential in more than 200 million children in the developing world. Lancet. 2007;369(9557):229–42. 17240290 10.1016/S0140-6736(07)60112-3

[33] Moreno LA, Rodríguez G. Dietary risk factors for development of childhood obesity. Curr Opin Clin Nutr Metab Care. 2007;10(3):336–41. 17414504 10.1097/MCO.0b013e3280a94f59

[34] Leung LC, Sung RY, So H-K, Wong SN, Lee KW, Lee KP, et al Prevalence and risk factors for hypertension in Hong Kong Chinese adolescents: waist circumference predicts hypertension, exercise decreases risk. Arch Dis Child. 2011;96(9):804–9. 10.1136/adc.2010.202770 21586437

[35] Yin HS, Johnson M, Mendelsohn AL, Abrams MA, Sanders LM, Dreyer BP. The health literacy of parents in the United States: a nationally representative study. Pediatrics. 2009;124(Supplement 3):S289–S98. 19861483 10.1542/peds.2009-1162E

[36] Sanders LM, Shaw JS, Guez G, Baur C, Rudd R. Health literacy and child health promotion: implications for research, clinical care, and public policy. Pediatrics. 2009;124(Supplement 3):S306–S14. 19861485 10.1542/peds.2009-1162G

[37] Drewnowski A, Darmon N. The economics of obesity: dietary energy density and energy cost. Am J Clin Nutr. 2005;82(1):265S–73S. 16002835 10.1093/ajcn/82.1.265S

[38] Ip P, Ho FKW, Louie HT, Chung TWH, Cheung YF, Lee SL, et al Childhood Obesity and Physical Activity-friendly School Environment. Journal of Pediatrics. Under review. 10.1016/j.jpeds.2017.08.017 28987751

[39] Adler NE, Newman K. Socioeconomic disparities in health: pathways and policies. Health Aff. 2002;21(2):60–76. 10.1377/hlthaff.21.2.60 11900187

[40] Boles M, Pelletier B, Lynch W. The relationship between health risks and work productivity. J Occup Env Med. 2004;46(7):737–45. 15247814 10.1097/01.jom.0000131830.45744.97

[41] The HKSAR Government. Half-yearly Economic Report 2012 Hong Kong: The HKSAR Government, 2013.

